# Intraocular Ossification

**Published:** 1871-08

**Authors:** F. B. Norcom

**Affiliations:** Chicago


A MONTHLY RECORD OF

*Medicine, Surgery, and the Collateral Sciences.*

EDITED BY J. ADAMS ALLEN, M.D., LL.D.; AND WALTER HAY, M.D.

---

VOL. XXVIII.—AUGUST, 1871.—No. 8.

---

### Original Communications.

ARTICLE I. — *Intraocular Ossification.* By F. B. NORCOM, M.D., Chicago.

Miss McC——; aged 23 years; nervo-sanguine temperament; of handsome, ruddy complexion, and fair physical development, showing the possession of exuberant health and sound constitution, though born, and, as we say hereabouts, "raised" in the State of Michigan (q. s.)

When about three years of age, was accidentally injured by a fragment from the stalk of a sunflower plant (*Helianthus annuus*), which was being used by her mother as a stimulant to the cutaneous surface, and to the soft, safe protuberances of a refractory brother, who, with an agility peculiar to youths under such circumstances, and with a natural disrelish for such applications, was ingeniously engaged in eluding and dodging the "raining blows," and with such benefit to himself, that, a neighboring table catching a spiteful stroke, a shower of splinters was sent in every direction, one of which perforated the cornea, and in all probability the capsule (and its lens), with partial dislocation from its normal attachments.

The entire foreign substance was removed very soon after the accident, and in a few hours violent local inflammatory action lighted up. Physicians were called in, but her fright and resistance were so urgent that, they could not draw the lids apart, and, as anæsthetics were not much in vogue at that period in the far West, nor their physiological action well understood, no further attempts were made at an examination, but the case was conducted upon general principles, and with such success that the entire cornea sloughed in three weeks, and the globe gradually emptied itself. The pain and suffering then began to abate, nor did the sound eye ever offer the slightest sympathetic disturbance.

In her seventeenth year she applied to Dr. J. B. Walker, an eminent ophthalmist of this city, for an artificial covering, though experiencing not the slightest subjective uneasiness in the stump, and this being nicely adapted, she returned home, well pleased with her improved appearance, and with full directions as to its removal and general management. This shell she wore pretty steadily for two and a half years, a period entirely too long, and with the usual result of an eroded and roughened surface, creating irritation, and finally inflammation of the palpebral conjunctiva, leading to a condition of fibro-sarcomatous granulation. As her stay in the city would be very limited, measures were immediately instituted to restrain inflammatory action and smooth down the granulations, at same time another shell inserted, and strict injunctions as to the treatment upon her return home. After this time, she paid several more visits to her surgeon, and always with the exchange of a new artificial eye, though the lid had not entirely regained its normal condition, nor become entirely free from slight bastard relapses. The first week in June she returned, having worn the last shell eighteen months, which, upon examination, was seen to have lost its glossy and smooth surface, and also with the addition to her troubles of intraocular irritation, and an enhanced crop of distended capillary loops of the upper lid, which were becoming daily more and more fibrous in character.

Her surgeon, feeling that the symptoms indicated a low grade of inflammation going on in the internal eye, and that its companion must soon take on symmetrical disease, and at the same time to facilitate his treatment of the lids, very properly advised extirpation, which was accordingly performed on the 9th of June.

The globe was reduced one-half its natural size, hard upon pressure, especially antero-posteriorly, the cornea gone, and its site occupied by a not very dense, translucent plate of fibro-plastic cicatricial tissue. It was partially opened by Dr. N. S. Barnes, who was present at the operation, and found to contain no fluid, but a hard substance occupying the hyaloid cavity. I had the honor to receive the specimen from the operating surgeon, Dr. Walker, with a request to examine it pathologically; and as it appeared to present rather a peculiar ossification of the internal structures, I have taken the liberty of making these few notes for the benefit of those fond of such investigations, especially for those studying ophthalmic surgery.

The incision was lengthened carefully, and the bulb well laid open. Size, one-half; cornea entirely disappeared, its place taken by the tissue above described; a few shrunken shreds of iris; sclerotic shriveled, but natural in structure; ciliary body gone, and ciliary ligament atrophic; choroid coat likewise, with, however, a light pigmentary layer; and the cavity occupied by an irregular osseous mass, composed of an anterior and posterior ovoid disk, joined by a delicate laminated column of bone. The posterior plate measures four lines in its transverse, and three and a half in its conjugage diameter; one line in thickness, gradually increasing to two at its junction with the column; begins at the entrance of the optic nerve, without any marked deficiency, and runs outwardly; is dense and serrated, and composed of bone structure chemically and anatomically. The column is one-half line in thickness, one in breadth, and three and a half in length, that is, from disk to disk; and is without question the coarcted and folded retina in a state of calcareous degeneration. The anterior disk is the shrunken capsule—the lens having been absorbed; concavo-convex, the convexity looking forward; fringed at its edge; fragile; cribriform; two and a half lines in its transverse, and two in its conjugate diameter; and is a perfect cretaceous transformation, effervescing with acids.

The weight of the entire body is 3½ grains; its posterior plate was adherent to the choroid, and, upon being detached, presented a quantity of pigmentary granules upon its under surface. A microscopical examination revealed laminated bone structure, with imperfectly developed lacunæ and canaliculi, but no wellmarked traces of Haversian canals, and no evidence of vascular endowment; though it must be confessed that, in subtracting a few particles for examination, I did it sparingly, for fear of injuring and defacing the beauty of the specimen. And as it lies now before me, so unique and instructive, I cannot refrain from making some few remarks, and mayhap indulging in some wayward speculations, for all of which I trust the reader will forgive me.

Lawson, in his admirable and practical treatise on "Injuries of the Eye, Orbit, and Eyelids," tells us, "that an eye which ceases to perform its function (ceases to see), whether from injury or disease, must in the course of time undergo disorganization, that is, become atrophic and degenerated, and the only exceptions are those whose corneas have been destroyed, as, for example, in purulent ophthalmias, without implicating the internal structures. Such eyes, of course, shrink from disuse, and remain quiet, seldom troubling their owners; but in those organs where the deeper-seated tissues are engaged, processes of change and degeneration ensue, the parts within waste more rapidly than the tunics of the globe, by shrinking, can accommodate themselves to, and the relative connection of the internal structures thus becomes disturbed." "From atrophy of the vitreous body, and diminished bulk, the retina, losing its support, falls forward separated from the choroid, and the intervening space becomes filled with an adventitious deposit, capable of being organized and developed into bone; while the retina becomes coarcted and folded upon itself like a cord," its properties as a vitalized tissue being completely lost. Every texture of the eye may be the seat of ossification, the result of changes induced by long-standing inflammation, and almost always of a traumatic nature, and all such changes begin with the vascular portions—as the ciliary body and the choroid coat—by effusions of plastic material, changing to fibrous tissue, next granular osseous deposits, and lastly bone structure.

Though it has been doubted by some, as to whether these calculous depositions possess the properties of bone, as vitalized and independent organizations, yet the researches of Hulke, Virchow, Stellwag and others, prove conclusively that they are moderately gifted with vascular canals, lacunæ, and canaliculi, and hence must perform certain and vital functions, otherwise it might be difficult to account for their growth and the superposition of various laminæ. Virchow evidently takes this view in his work on "Morbid Tumors," as quoted in the July number (1870) of *The American Journal of Medical Sciences*, where two interesting cases of bony deposit within the eye are introduced, by Dr. Harlan, to the March meeting of the Pathological Society, and a careful microscopical examination of both specimens by Dr. W. W. Keen. Stellwag says, too, "The ossification of these new formations begins from the external layers, while new connective tissue layers are placed on the inner surface."

Very few of the writers believe that the retina ever takes on this species of degeneration. Mackenzie doubts it, following, however, the doctrine of Panizza, who, from his dissection of a case, quoted in that learned author's work, page 649, merely considers such appearances of the hyaloid membrane and retina as calcareous incrustations deposited by the condensation of extravasated fluids; while Virchow, as above noticed, believes that the osteomata are small flat bones, developed on the internal vascular tunic, always the result of chronic choroiditis, and have been mistaken for ossifications of the retina. Stellwag, on the other hand, says, "that if any part of the retina remains in contact with the bony plates, it may also become bony." With this specimen in my hand, and from a careful examination of its position and peculiar laminated appearance, I cannot doubt but that the stem, joining the disks, is the retina transformed into bone, not by incrustations deposited upon it as a *point d'appui*, but by the same right, and in the same manner, as other vitalized tissues have to take on such changes; nor does the fact of its degenerated condition, with all its vessels atrophied, and separated from its choroid, materially alter the question in any respect, as it is by no means necessary in such calcareous degenerations that the substances have an independent vascular system, or have connection with parts so endowed (Rokitausky), and a very good illustration is furnished in the case of free bodies undergoing such transformations enclosed in serous sacks or cavities. Happy examples of changes from fibrous to osseous material will be seen in the formation of phlebolithes from clots of blood; of branching and irregular pieces of bonelike matter found in obliterated veins; and, lastly, of mortar grains in the fibrous deposits on the heart's valves (Paget).

In these remarks I have only touched lightly and incidentally the several points of interest; first, the conversion of bone from ordinary inflammatory exudations, and the various processes of change; second, the ossification of the retina, for in this case no other portion of the internal eye could have furnished the connecting column; and, lastly, ossific degeneration of substances without vascular supply of their own, or dependence on other structures so supplied.

